# Stiffer Bonding of Armchair Edge in Single‐Layer Molybdenum Disulfide Nanoribbons

**DOI:** 10.1002/advs.202303477

**Published:** 2023-09-11

**Authors:** Chunmeng Liu, Kenta Hongo, Ryo Maezono, Jiaqi Zhang, Yoshifumi Oshima

**Affiliations:** ^1^ Henan Key Laboratory of Diamond Optoelectronic Materials and Devices Key Laboratory of Materials Physics Ministry of Education and School of Physics & Microelectronics Zhengzhou University Zhengzhou 450052 China; ^2^ School of Materials Science Japan Advanced Institute of Science and Technology 1‐1 Asahidai Nomi Ishikawa 923‐1292 Japan; ^3^ Center of Advanced Analysis & Gene Sequencing Zhengzhou University Zhengzhou 450001 China; ^4^ Research Center for Advanced Computing Infrastructure Japan Advanced Institute of Science and Technology Nomi Ishikawa 923‐1292 Japan; ^5^ School of Information Science Japan Advanced Institute of Science and Technology Nomi Ishikawa 923‐1292 Japan; ^6^ Institute of Quantum Materials and Physics Henan Academy of Sciences Zhengzhou 450046 China

**Keywords:** edge effect, nanoribbons, single‐layer molybdenum disulfide, transmission electron microscopy, width‐dependent Young's modulus

## Abstract

The physical and chemical properties of nanoribbon edges are important for characterizing nanoribbons and applying them in electronic devices, sensors, and catalysts. The mechanical response of molybdenum disulfide nanoribbons, which is an important issue for their application in thin resonators, is expected to be affected by the edge structure, albeit this result is not yet being reported. In this work, the width‐dependent Young's modulus is precisely measured in single‐layer molybdenum disulfide nanoribbons with armchair edges using the developed nanomechanical measurement based on a transmission electron microscope. The Young's modulus remains constant at ≈166 GPa above 3 nm width, but is inversely proportional to the width below 3 nm, suggesting a higher bond stiffness for the armchair edges. Supporting the experimental results, the density functional theory calculations show that buckling causes electron transfer from the Mo atoms at the edges to the S atoms on both sides to increase the Coulomb attraction.

## Graphical abstract

The edges of nanoribbons have unique electronic and spin states due to the lack of symmetry. It is found that the Young's modulus of single‐layer molybdenum disulfide nanoribbons with armchair edges increases with decreasing the width below 3 nm. It means that the bond strength is stiffer at the armchair edge. Density functional theory calculations explain this by buckling at the edge.

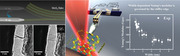

## 1 Introduction

The nanoribbons of 2D materials are expected to exhibit unanticipated functionalities due to their unique electronic, mechanical, and optical properties. Research on these nanoribbons has actually been vigorously pursued from fundamental understanding to application as new devices are being devised using the nanoribbons of graphene, molybdenum disulfide (MoS_2_), etc.^[^ ^1^, ^2^, ^3^, ^4^ ^]^ Nanoribbon edges have recently attracted attention because they produce unique physical and chemical properties, such as catalytic reactions, optical response, anisotropic thermal conductivity, and local magnetization.^[^ ^5^, ^6^, ^7^, ^8^, ^9^, ^10^, ^11^, ^12^, ^13^, ^14^, ^15^ ^]^ They influence the electronic structure of a nanoribbon, such that graphene nanoribbons with armchair edges have a band gap caused by quantum confinement, while those with zigzag edges have a band gap caused by spin polarization at the edges.^[^ ^16^, ^17^ ^]^ These edges also influence the mechanical response of nanoribbons.^[^ ^18^, ^19^, ^20^, ^21^ ^]^ However, very few experimental studies have been conducted to reveal the bond stiffness at the edge due to technical difficulties, as the bond stiffness must be precisely measured while simultaneously observing the atomic structure to evaluate the bond stiffness at the edge.

MoS_2_ nanoribbons attract much attention because of their high chemical stability and stiffness, intrinsic band gap, and other characteristics.^[^ ^22^, ^23^ ^]^ These nanoribbons, which are less than a few atoms thick, are expected for use in the channel layers of flexible electronic devices and sensor resonators because of their high mechanical strength and flexibility.^[^ ^24^, ^25^, ^26^, ^27^ ^]^ The high Young's modulus of MoS_2_ nanoribbons is suitable for design and performance optimization in various applications.^[^ ^28^, ^29^, ^30^, ^31^ ^]^ The single‐layer MoS_2_ (SLMoS_2_) has also been reported to have a band gap that can be tuned by tensile strain.^[^ ^32^, ^33^, ^34^ ^]^ Through the first‐principles calculations, the SLMoS_2_ bandgap is predicted to decrease as the tensile strain increases,^[^ ^35^ ^]^ such that the transition from direct to indirect gap occurs at 0.01 strain, and that from semiconductor to metal occurs at 0.10 strain.^[^ ^36^ ^]^ In short, SLMoS_2_ tunes its electronic and optical properties through mechanical deformation. Considering that it is expected to be utilized for fabricating transistors that exhibit extremely high on/off ratios and very low power dissipation,^[^ ^37^, ^38^, ^39^, ^40^, ^41^ ^]^ the mechanical properties of SLMoS_2_ must be clearly understood.

The elastic modulus of SLMoS_2_ has been extensively investigated through atomic force microscopy (AFM).^[^ ^42^, ^43^, ^44^, ^45^, ^46^, ^47^, ^48^ ^]^ In one study, an exfoliated SLMoS_2_ was transferred onto a patterned substrate containing a series of circular holes. Its mechanical properties were then measured by nanoindentation based mainly on AFM. In 2011, Betolazzi et al.^[^ ^42^ ^]^ estimated the effective modulus of SLMoS_2_ as 270 ± 100 GPa through AFM indentation tests. Their value was in a good agreement with the 210 GPa Young's modulus predicted using the first‐principles density functional theory (DFT) calculations reported by Copper et al.^[^ ^49^ ^]^ in 2013. The Young's modulus, breaking strength, and friction coefficient of SLMoS_2_ can be measured by AFM indentation tests. However, the MoS_2_ film prestretching caused by the internal strain between the layer and the substrate induced in the transfer process should be considered because it may lead to a large error of the measured Young's modulus.^[^ ^50^ ^]^ In addition, the uniaxial elastic modulus could not be measured because the AFM probe was pressed at the center of the suspended SLMoS_2_ in the circular hole. Thus, the size and the structure dependence of the Young's modulus of SLMoS_2_ nanoribbons could not be obtained through the AFM nanoindentation tests.

The size and the edge‐structure dependence of the Young's modulus of SLMoS_2_ nanoribbons have mainly been investigated using a molecular dynamics simulation. Jiang et al.^[^ ^51^ ^]^ reported that the Young's modulus for both zigzag‐ and armchair‐edge SLMoS_2_ (Arm‐SLMoS_2_) decreased with the width decrease. However, Bao et al.^[^ ^52^ ^]^ reported an opposite tendency and showed that the elastic modulus of the Arm‐SLMoS_2_ increased as the width became narrower. These conflicting theoretical calculation results required an experimental study.

An advanced nanomechanical measurement was recently established by developing an in situ transmission electron microscope holder equipped with a quartz‐length extension resonator (LER).^[^ ^53^, ^54^, ^55^, ^56^ ^]^ The LER made from quartz with a high Young's modulus has a high resonant frequency of ≈1 MHz due to its elongated shape, which is effective in noise reduction. It also has a high Q‐factor that can sufficiently suppress the dissipated energy during the measurements, resulting in a high accuracy. The equivalent spring constants of the nanoribbon can be measured as at least one order of magnitude higher in accuracy by the LER compared to the conventional Si cantilever. The in situ transmission electron microscopy (TEM) observation also showed the possibility of identifying the structure of the ultra‐narrow nanoribbon, which is suitable for estimating the edge contribution. The size and the shape of nanocontacts (NCs) and their supporting bulk parts are determined by TEM observations. Hence, the Young's moduli of gold (Au)^[^ ^55^ ^]^ or platinum (Pt)^[^ ^56^ ^]^ NCs were previously precisely measured by removing the contribution of the bulk parts from the experimental data. We thought that this nanomechanical measurement meets the requirements necessary for measuring the bond stiffness at the nanoribbon edges.

In this work, we precisely measure the bond stiffness of the MoS_2_ nanoribbon edges. To the best of our knowledge, this is the first work to perform a precise measurement of the width‐dependent Young's modulus of the Arm‐SLMoS_2_ nanoribbon through an in situ TEM observation using the transmission electron microscope holder equipped with an LER. Our experimental results show that the Young's modulus of Arm‐SLMoS_2_ is inversely proportional to the width, indicating that the armchair‐edge bonding is stiffer than the interior. These experimental results are explained by the buckling of the Mo─S bonding at the armchair edge during the DFT calculations.

## 2 Results and Discussion

### 2.1 Preparation and Characterization of Multilayer MoS_2_ Flakes

**Figure 1:**
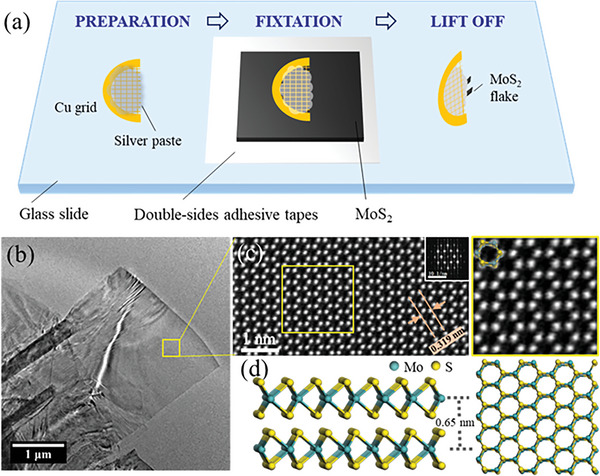
Multilayer MoS_2_ sample preparation and characterization: a) schematic illustration of the MoS_2_ flake preparation; b) TEM image of a suspended multilayer MoS_2_ flake; c) ADF‐STEM and higher‐magnification (yellow square) images of the MoS_2_ flake with a white atom contrast showing a 0.319 nm lattice constant (inset: corresponding fast‐Fourier transformation pattern); and d) side and front views of the multilayer MoS_2_ flake model, with the adjacent layers stacking from a 60° rotation. The measured layer spacing was 0.65 nm.

The samples for the in situ TEM observation were prepared as shown in **Figure** **1**a. First, a 200‐mesh TEM grid was cut in half and coated with conducting silver (Ag) paste on the grid bars. Second, a block of natural MoS_2_, which was a few hundred micrometers thick, was adhered to a double‐sided adhesive tape on a glass slide and repeatedly peeled off to thin it using Scotch tape. Third, the prepared half TEM grid coated with Ag paste was adhered to a MoS_2_ sheet. The half TEM grid was removed with a tweezer once the Ag paste was cured. Accordingly, the MoS_2_ flakes with a layer thickness ranging from a few to tens of layers remained at the half TEM grid edge. Finally, the prepared TEM grid was adhered to a copper plate fixed at the head of our homemade TEM holder.

The quality of the natural MoS_2_ block was characterized via X‐ray diffraction, in which the peak positions indicated the MoS_2_‐2H structure (Figure S1, Supporting Information ). The size and the quality of the exfoliated MoS_2_ flake were further evaluated using the 200 kV transmission electron microscope, JEM‐ARM200F. The suspended MoS_2_ flake had a rectangular shape with 3.5 µm length and 2.8 µm width (Figure 1b). The annular dark‐field scanning TEM (ADF‐STEM) images and the corresponding fast‐Fourier transformation (FFT) pattern (Figure 1c) depicted that the flake had a highly crystalline and multilayer structure. The lattice constant estimated from an ADF‐TEM image was ≈0.32 nm, which was consistent with the MoS_2_ structure (a = 0.312 nm) (Figure 1d). Considering that the flakes are often folded at the edges, the layer number of the MoS_2_ flake was determined from the folded flake edge in the TEM image. In Figure S2 (Supporting Information), the MoS_2_ flake shows eight clear parallel dark lines indicating eight layers. The measured spacing of these eight dark lines was 0.65 nm, which matched with the bulk MoS_2_ structure spacing in Figure 1d.

### 2.2 Fabrication and Observation of the Single‐Layer MoS_2_ Nanoribbons

The SLMoS_2_ nanoribbon was produced by peeling the outermost layer of the folded edge of a multilayer MoS_2_ flake by approaching the tungsten (W) tip (Movie S1, Supporting Information). The flake edges were often folded.^[^ ^57^, ^58^, ^59^ ^]^ The space between the layers at the outer sides of the folded MoS_2_ flake increased, making it easier for the layers to separate from each other.^[^ ^60^ ^]^ This suggests that the outermost layer of the folded MoS_2_ flake can be peeled off as SLMoS_2_ by the W tip.

**Figure 2:**
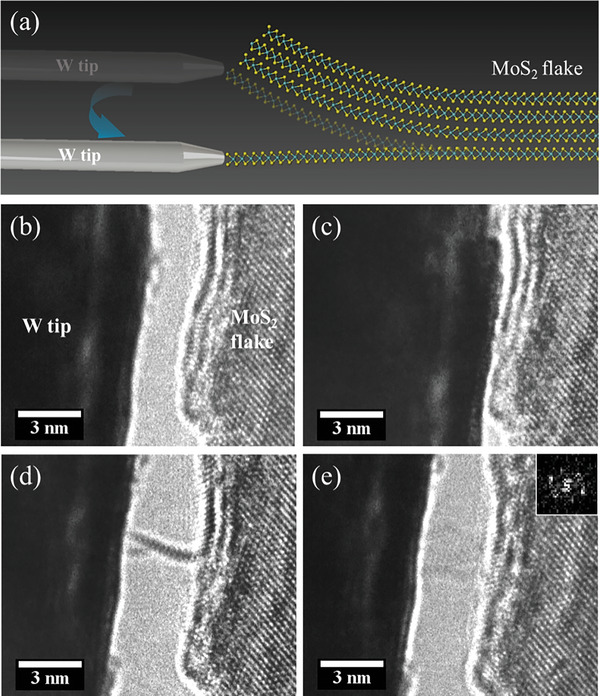
Fabrication process of the single‐layer MoS_2_ nanoribbons: a) schematic of the fabrication method of the SLMoS_2_ nanoribbon from a multilayer MoS_2_ flake during TEM and b–e) TEM images captured at the time sequence from Movie S1 (Supporting Information) showing the fabrication process.

**Figure** **2**a illustrates the peel‐off process. First, the edge of the small multilayer MoS_2_ nanoflakes was identified through the TEM images in Figure 2b. These small SLMoS_2_ nanoflakes may be formed during the exfoliation process of the MoS_2_ block. Next, the W tip was moved toward the edge of the multilayer MoS_2_ nanoflakes and attached to their outermost single layer (Figure 2c). The W tip was moved at an inclined angle after making the contact. Consequently, SLMoS_2_ followed the W tip and was gradually peeled off from the multilayer MoS_2_ flakes (Figure 2d). Finally, the SLMoS_2_ nanoribbon was fabricated, and the orientation was tilted to identify its width and edge structures by slightly tuning the W tip position (Figure 2e). Supporting Information Movie S1 shows the whole fabrication process of the SLMoS_2_ nanoribbons. The number of layers was confirmed by the side‐view TEM images of the SLMoS_2_ nanoribbons in Figure S3 and S4, Movie S2 and S3 (Supporting Information).

However, the observation showed that SLMoS_2_ above the 10 nm width was hardly pulled off. We think that the outermost monolayer may have had defects, including cracks,^[^ ^47^, ^61^ ^]^ in the layer during the flake folding process, which resulted in a thin nanoribbon that became a part of the layer when it was pulled out with the W tip. If no defects were produced, pulling out with the W tip would have been difficult because it was strongly adsorbed to the flake body. Choosing the folded MoS_2_ flake and controlling the peeling direction of the W tip made it possible to fabricate the armchair‐ or zigzag‐edge SLMoS_2_ nanoribbons (Figure S5, Supporting Information). Few‐layer nanoribbons can also be fabricated, as depicted in Figure S6 and Movie S4 and S5 (Supporting Information). However, at the present status, we cannot precisely give the mechanical parameters for fabricating MoS_2_ nanoribbons with a specified number of layers. We will further analyze our data and try to clarify the experimental parameters for preparing different MoS_2_ nanoribbon layers in a future work. In this study, we focused on the Arm‐SLMoS_2_ nanoribbons with widths ranging from 5.15 to 1.13 nm to investigate the effect of the edge or surface on the mechanical properties of the MoS_2_ nanoribbons.

### 2.3 In Situ TEM Experiment on the Single‐Layer MoS_2_ Nanoribbons

**Figure 3:**
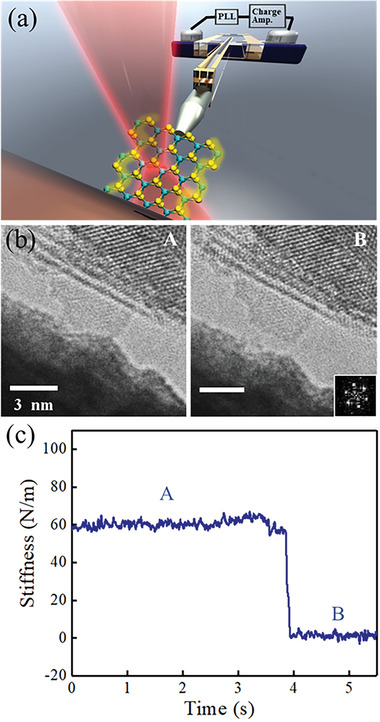
a) Schematic illustration of the in situ TEM experiment on the SLMoS_2_ nanoribbon. b) TEM images captured during (A) and after (B) the stiffness measurement. The top side of the TEM images depicts a multilayer MoS_2_ flake with a peeled‐off SLMoS_2_ nanoribbon. The bottom side displays the W tip used to make contact with the SLMoS_2_ nanoribbon. The lower‐right image shows the corresponding FFT pattern. c) Typical variations in the SLMoS_2_ nanoribbon stiffness during the measurement. A and B correspond to the captured moments in (b).

The in situ TEM experiment on the SLMoS_2_ nanoribbon was set up as shown in **Figure** **3**a. A quartz LER was used to estimate the Young's modulus of the MoS_2_ nanosheets (Figure S4, Supporting Information). The W tip was made to approach and establish contact with the ≈3.7 nm‐wide SLMoS_2_ nanoribbon for the stiffness measurement. The TEM image in Figure 3bA illustrates that the W tip made contact with the SLMoS_2_ nanoribbon. They were separated in the TEM image in Figure 3bB when the W tip was pulled back (Movie S6, Supporting Information). This SLMoS_2_ nanoribbon revealed armchair‐edge structures that can be identified by a reciprocal lattice spot in the FFT pattern shown in Figure 3bB.

In Figure 3c, the nanoribbon stiffness was simultaneously measured through the TEM observation. The average stiffness was found to be ≈60 N m^−1^ when the W tip came into contact with the nanoribbon in Stage A. It became 0 when the W tip was removed from the nanoribbon in Stage B.

**Figure 4:**
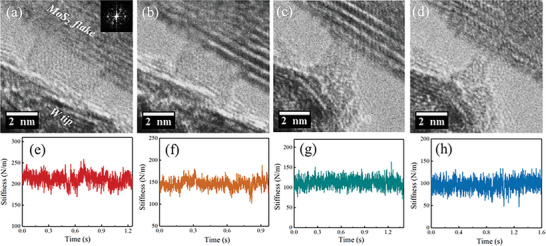
a–d) TEM images and e–h) corresponding measured stiffness of the armchair‐edge SLMoS_2_ nanoribbons with 5.15, 3.85, 2.14, and 1.13 nm widths. The TEM image tended to blur as the nanoribbon became thinner due to the mechanical vibration caused by the noise from the voltage applied to the piezo.

**Figure** **4** exhibits the TEM images of the Arm‐SLMoS_2_ nanoribbons with four different widths of 5.15, 3.85, 2.14, and 1.13 nm. The nanoribbons in Figure 4a–d were fabricated by peeling off the outermost layer from the flakes along the direction parallel to the armchair edge to make an armchair‐edge single‐layer nanoribbon. These armchair edges were confirmed by the corresponding FFT patterns showing that the nanoribbon axis was parallel to the {100} reciprocal lattice vector (Figure S8, Supporting Information). Each nanoribbon configuration seemed stable without obvious defects because neither the shape and contrast in the TEM images nor the time evolution of the stiffness changed (Figure 4e–h).

The irradiation damage for the nanoribbons seemed negligible.^[^ ^62^ ^]^ In the case of the 2D materials, the knock‐on damage caused by electron irradiation makes vacancy or structural changes at acceleration voltages above 100 kV. However, this damage will practically be recovered at a temperature higher than room temperature.^[^ ^63^ ^]^ In this experiment, we supposed that the temperature may be raised by current annealing with a 10 mV bias voltage to maintain the original structure. The corresponding average stiffness was measured as 211, 146, 113, and 97 N m^−1^. The Young's modulus of 22 Arm‐SLMoS_2_ nanoribbons with different widths were also obtained, including these four nanoribbons.

### 2.4 Young's Modulus of the Armchair‐Edge Single‐Layer MoS_2_ Nanoribbons

Note that the measured stiffness values (*k_m_*) include contributions from the Arm‐SLMoS_2_ nanoribbon (*k_ribbon_*), the MoS_2_ flake (eight layers in thickness) connected with the nanoribbon (*k_flake_*), and the W tip (*k_W_*). The measured stiffness (*k_m_*) is a series coupling of these three stiffness values and expressed as follows:

(1) $$\frac{1}{k_{m}}=\frac{1}{k_{ribbon}}\;+\frac{1}{k_{flake}}+\frac{1}{k_{W}}$$

Precisely estimating the Young's modulus of the Arm‐SLMoS_2_ nanoribbon required removing the contributions of the MoS_2_ flake and the W tip supporting the Arm‐SLMoS_2_ nanoribbon from the measured stiffness.

We estimated the MoS_2_ flake stiffness by its dimension. The flake had length, width, and thickness of 3.5 µm, 2.8 µm, and 5.2 nm (eight layers), respectively (Figure 1b and Figure S2, Supporting Information). The Young's modulus of the suspended MoS_2_ nanosheets with five to 25 layers was 330 ± 70 GPa.^[^ ^50^ ^]^ Hence, the MoS_2_ flake (*k_flake_*) stiffness was calculated as 1373 N/m as follows:^[^ ^55^ ^]^

(2) $$k\;=\;Y\frac{w\cdot d}{L}$$

where *Y*, *w*, *L*, and *d* correspond to the material's Young's modulus, width, length, and thickness, respectively. The stiffness of the W wire (*k_W_*), including the connection part with the MoS_2_ nanoribbon, was calculated on the order of 10^5^ N m^−1^. Figure S9 and Table S1 and S2 (Supporting Information) present the calculation details. The W wire tip cut using pliers was not as sharp as that made by chemical etching. The aspect ratio of the length to the wire diameter at the connection part with the nanoribbon (Figure S9, Supporting Information) was small. Therefore, the *k_W_* inverse could be ignored in Equation (1).

**Figure 5:**
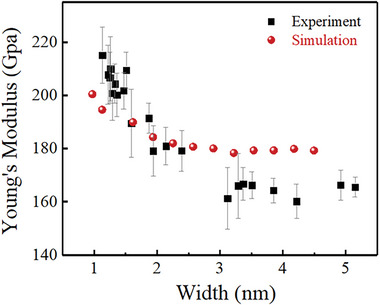
Width dependence of the Young's modulus of the Arm‐SLMoS_2_ nanoribbons. The black squares with an error bar represent the experimentally measured value. The red spheres indicate the simulated value by the DFT calculations (Supporting Information Section S8 and S9).

The Arm‐SLMoS_2_ nanoribbon stiffness was obtained by removing the MoS_2_ flake contribution (*k_flake_* = 1373 N m^−1^). The Young's modulus of the nanoribbon was estimated using Equation (2), in which the length was 2 nm, and the widths were 5.15, 3.85, 2.14, and 1.13 nm, as depicted in the TEM images in Figure 4. **Figure** **5** displays the Young's modulus of the nanoribbon, which was inversely proportional to the width of the Arm‐SLMoS_2_ nanoribbon below 3 nm. The Young's modulus increased from 179 ± 8 to 215 ± 11 GPa as the width decreased from 2.39 to 1.13 nm. By contrast, it was almost constant ≈165 GPa above the 3 nm width. The values in the present results were slightly lower than those obtained from the previous studies (i.e., 270 ± 100 GPa^[^ ^42^ ^]^ and 185 ± 46 GPa^[^ ^49^ ^]^) that performed the AFM indentation tests. The differences from our results can be attributed to the different measurement methods. This is considering that the Young's modulus was estimated under biaxial tensile stress during the AFM indentation tests and measured under uniaxial stress along the armchair edges in this work. Akhter et al.^[^ ^64^ ^]^ and Hung et al.^[^ ^65^ ^]^ pointed out that the experimental results for the biaxial elastic modulus in the AFM indentation tests were higher than the simulation results for the uniaxial elastic modulus. The Young's modulus of SLMoS_2_ under uniaxial tension was previously only reported in theoretical calculations^[^ ^51^, ^66^, ^67^, ^68^ ^]^ due to experiment difficulty. To the best of our knowledge, we are the first to report on the experimental results of the width‐dependent Young's modulus of the Arm‐SLMoS_2_ nanoribbons under uniaxial tensile stress. Note that the Young's modulus measurements for Arm‐SLMoS_2_ nanoribbons of different lengths almost showed the same width dependence for the nanoribbons of 2 nm length (Figure S10, Supporting Information). Thus, the length of the nanoribbon did not matter for the obtained width dependence of Young's modulus.

The DFT calculations for the Arm‐SLMoS_2_ nanoribbon were performed to understand the reason behind the width‐dependent Young's modulus. Since the length of the nanoribbon did not matter for the obtained width dependence of Young's modulus, we assumed infinite periodicity in the length direction. In these calculations, a supercell with a sufficiently wide area perpendicular to the nanoribbon axis with 50 Å width and 20 Å thickness was prepared to verify the edge effects. Assuming a uniform distortion, the size of the supercell along the nanoribbon axis direction was changed from 5.363 to 5.603 Å at a 0.02 Å step to evaluate the stiffness. The stiffness was obtained from a second‐order derivative of the calculated energy with respect to the strain at the minimum. The Young's modulus was evaluated by considering the Arm‐SLMoS2 nanoribbon dimensions (Supporting Information Section S8 and S9).

Figure 5 shows that the calculated Young's modulus of the Arm‐SLMoS_2_ nanoribbons, which is represented by red sphere in the figure, increases with the decreasing width, indicating the same tendency with the experimental results. The calculated Young's modulus decreased from 201 to 180 GPa as the width increased from 0.97 to 2.89 nm. It remained constant at ≈179 GPa for the width above 2.89 nm. Considering the edge atom‐to‐internal atom ratio with a decreasing width, both the experimental and calculated size‐dependent Young's moduli may be explained by the edge effect.^[^ ^69^, ^70^, ^71^ ^]^ The measured Young's modulus was slightly lower than the calculated one when the nanoribbons were wider than 3 nm. However, the ultra‐narrow nanoribbons with a width below 3 nm showed a Young's modulus that was similar to the theoretical one, suggesting defect reduction. This result was in agreement with the previous works showing less defects for narrow nanoribbons.^[^ ^47^, ^61^ ^]^

### 2.5 Interpretation of the Width‐Dependent Young's Modulus

**Figure 6:**
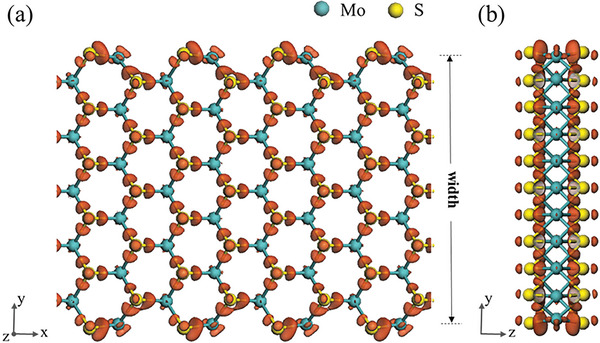
Top a) and side b) views of the deformation electron density isosurfaces of the Arm‐SLMoS_2_ nanoribbon with a finite width (four or five rings). The top view shows the x–y plane. The side view depicts the y–z cross‐sections. The blue spheres in the model represent the Mo atoms, while the yellow ones represent the S atoms. The brown closed surfaces depict the 7 × 10^−2^ e Å^−3^ electron density isosurfaces. Note that SLMoS_2_ comprises S, Mo, and S layers with different heights.

**Figure** **6** shows the charge distribution of the Arm‐SLMoS_2_ nanoribbon obtained through the DFT calculations. The electron density isosurfaces of 7 × 10^−2^ e Å^−3^, which are indicated by brown closed surfaces in this figure, depicted that more electrons were accumulated in the edge S atoms of the Arm‐SLMoS_2_ nanoribbon than at the interior S atoms. This result suggests that electrons can be transferred from the edge Mo atom of the Arm‐SLMoS_2_ nanoribbon to the S atoms on both sides. Optimized geometry confirmed that the edge Mo atom was buckled. Dimerization is known to form a 2 × 1 reconstruction on the Si (001) surface.^[^ ^72^ ^]^ The dimer is further reduced in energy when one Si atom is buckled to take on an asymmetric atomic configuration. This is attributed to the buckling eliminating the s‐ and p‐orbital hybrid and creating an s‐like state in the atom displaced toward the surface while forming a p‐like state in the atom displaced toward the substrate, such that the electrons move to the atoms displaced to the surface side. As an analogy to this asymmetric dimer, we think that buckling caused the electrons to be transferred from the edge Mo ion to the S ion, albeit the bonding between the Mo and S ions being a mixture of ionic and covalent natures.^[^ ^73^ ^]^

The DFT calculations obtained a 2.31 Å Mo─S bond length at the edge and 2.43 Å in the interior, which were consistent with the results of the previous studies.^[^ ^74^ ^]^ The calculated Mulliken charges of the edge Mo and S atoms indicated by Mo_1_ and S_7,8_ in Figure S13 (Supporting Information) were 0.39 and −0.20, respectively. The charge difference between the edge Mo and S atoms were obviously larger than that between the internal Mo (0.23, as indicated by Mo_2_–Mo_6_ in Figure S13, Supporting Information) and S (−0.11 to −0.12, as indicated by S_9,10_–S_17,18_ in Figure S13, Supporting Information) atoms. These larger Mulliken charges of the edge S atoms compared to the interior S atoms (0.160) were almost the same as the reduced amounts of the edge Mo atoms compared to the interior Mo atoms (0.157). In other words, the charge was mainly transferred from the edge Mo atoms to the edge S atoms, which was consistent with the result in Figure 6. The Coulomb attraction in the Mo–S bond at the edge may be greater than that in the Mo─S bond inside the nanoribbon, and the Mo─S covalent interaction may have been reduced by the charge transfer at the edge. The Mo─S covalent interaction may be enhanced by the unsaturated bond of the edge S atoms. The edge Mo and S ions of the Arm‐SLMoS_2_ nanoribbon were easily displaced by their low coordination number; hence, we think that the Coulomb attraction shortened the bond length and enhanced the stiffness.

With the above discussion, we may conclude that the Mo─S bond at the edge can be stiffer than that in the interior. The smaller the nanoribbon width, the greater the substantial edge effect provided, which is consistent with the experimental result that the width dependence of Young's modulus is more pronounced below 3 nm width. The ratio of the edge atoms, including both the edge atoms of the four and five rings, to the interior atoms was approximately 21% when the width was narrower than ≈3 nm (corresponding to nine six‐membered rings in width), which was a non‐negligible value.

## 3 Conclusion

The width dependence of Young's modulus for an armchair‐edge single‐layer MoS_2_ (Arm‐SLMoS_2_) nanoribbon was investigated herein through in situ TEM observation, which allowed us to obtain its structural information while simultaneously measuring its stiffness. Arm‐SLMoS_2_ nanoribbons can be fabricated by peeling the outermost MoS_2_ layer from the folded MoS_2_ flake. The Young's modulus of the Arm‐SLMoS_2_ nanoribbon used in this work was precisely estimated by removing the contributions of the flake and the W tip, thereby supporting the nanoribbon from the measured stiffness. The Young's modulus had an inversely proportional relationship with the width of the Arm‐SLMoS_2_ nanoribbon. That is, the Young's modulus almost remained constant ≈166 GPa when the ribbon width was wider than 3 nm. The Young's modulus clearly increased from 179 to 215 GPa when the ribbon width decreased from 2.4 to 1.1 nm. This dependence was well reproduced by the DFT calculations revealing that the Mo─S bonds at the armchair edge were stiffer than those at the interior due to buckling. The edge effect enhanced and dominated the Young's modulus of the armchair‐edge SLMoS_2_ nanoribbon as the width decreased, especially at a width smaller than ≈3 nm. In conclusion, the edges play an important role in the mechanical properties of SLMoS_2_ nanoribbons.

## 4 Experimental Section

### Developed TEM Holder and Measurement System

Figure S7(a) (Supporting Information) depicts the head part (sample stage) of developed in situ TEM holder. The left side of the figure shows that the prepared MoS_2_ flakes were fixed on the copper plate, while the right side illustrates a 10 µm‐diameter tungsten (W) tip attached to the end of the LER with Ag paste. The W tip position was controlled to approach and establish contact with the MoS_2_ flake edge using a compact ultrasonic linear motor (TULA50, Technohands) (coarse motion) and a tube piezo (fine motion).

Figure S7(b) (Supporting Information) shows the stiffness measurement system. The LER was induced to oscillate at its resonance frequency (f_0_) by applying an excitation voltage to one of its electrodes (blue color, Figure S7, Supporting Information). The MoS_2_ nanoribbon stiffness (*k*) was obtained from the resonance frequency shift (∆f), as shown by formula *k* ≈ 2 × *k*_0_(∆*f*/*f*_0_). The resonance frequency was determined by the total stiffness corresponding to the serial coupling of the LER stiffness and the stiffness of the sample that came into contact with the LER (frequency modulation method).^[^ ^75^ ^]^   Supporting Information Section S4 provides details on the measurement methods.

### Cleaning

Prior to the in situ TEM experiment, the sample mounted in the TEM holder was baked at ≈100 °C for at least 24 h in a vacuum chamber to remove the contamination from the prepared multilayer MoS_2_ flake as much as possible.

### TEM Observation

High‐resolution TEM observations were conducted using an ultra‐high vacuum transmission electron microscope (JEM‐2000VF) with 200 kV accelerating voltage at room temperature. The ultra‐high vacuum conditions (≈1 × 10^−6^ Pa) inside the TEM column were effective in avoiding contamination and gas adsorption onto the sample. The TEM images were captured by a charge‐coupled device camera at 0.2 s intervals while the stiffness was being simultaneously measured.

## Conflict of Interest

The authors declare no conflict of interest.

## Author Contributions

Y.O. conceived and supervised the entire research. J.Z. and C.L. conceived the overall study, developed the in situ TEM holder, performed measurements, and analyzed data. K.H. and R.M. assisted in first‐principles calculation. Y.O., J.Z., and C.L. wrote the manuscript, with all the authors contributing to the discussion and revision of the manuscript. All authors have approved the final version of the manuscript.

## Supporting information

Supporting Information

Supplemental Movie 1

Supplemental Movie 2

Supplemental Movie 3

Supplemental Movie 4

Supplemental Movie 5

Supplemental Movie 6

## Data Availability

Research data are not shared.
