# Supplementary material for: Stiffer Bonding of Armchair Edge in Single‐Layer Molybdenum Disulfide Nanoribbons
Source: Adv Sci (Weinh). 2023 Sep 11;10(30):2303477. doi: 10.1002/advs.202303477 (PMC10602518; doi:10.1002/advs.202303477)
Supplement: Supplementary file 1 — Supporting Information [file ADVS-10-2303477-s006.pdf]

ADVANCED SCIENCE
Open Access

Supporting Information

for *Adv. Sci.*, DOI 10.1002/advs.202303477

Stiffer Bonding of Armchair Edge in Single-Layer Molybdenum Disulfide Nanoribbons

Chunmeng Liu, Kenta Hongo, Ryo Maezono, Jiaqi Zhang and Yoshifumi Oshima**

ADVANCED SCIENCE
Open Access

Supporting Information

for *Adv. Sci.*, DOI 10.1002/advs.202303477

Stiffer Bonding of Armchair Edge in Single-Layer Molybdenum Disulfide Nanoribbons

Chunmeng Liu, Kenta Hongo, Ryo Maezono, Jiaqi Zhang and Yoshifumi Oshima**

Supporting information

Stiffer bonding of armchair edge in single layer molybdenum disulfide nanoribbons

Chunmeng Liu[a,b,c], *Kenta Hongo*[d], *Ryo Maezono*[e], *Jiaqi Zhang*[a,b,f,*], *Yoshifumi Oshima*[b,*]

a. Henan Key Laboratory of Diamond Optoelectronic Materials and Devices, Key Laboratory of Materials Physics, Ministry of Education, and School of Physics & Microelectronics, Zhengzhou University, Zhengzhou 450052, China
b. School of Materials Science, Japan Advanced Institute of Science and Technology, 1-1 Asahidai, Nomi, Ishikawa 923-1292, Japan
c. Center of Advanced Analysis & Gene Sequencing, Zhengzhou University, Zhengzhou 450001, China
d. Research Center for Advanced Computing Infrastructure, Japan Advanced Institute of Science and Technology, Nomi, Ishikawa 923-1292, Japan
e. School of Information Science, Japan Advanced Institute of Science and Technology, Nomi, Ishikawa 923-1292, Japan
f. Institute of Quantum Materials and Physics, Henan Academy of Sciences, Zhengzhou 450046, China

[*]Corresponding author: zhangjq@zzu.edu.cn, oshima@jaist.ac.jp

Content

S1. X-ray diffraction of natural MoS_2 block

S2. TEM image of prepared MoS_2 flake

S3. Fabrication of the single-layer MoS_2 nanoribbon in TEM

S4. Developed TEM holder and measurement system

S5. Orientation analysis of MoS_2 nanoribbon by FFT pattern

S6. Estimation of the stiffness of MoS_2 flake and W tip

S7. Young's modulus of Arm-SLMoS_2 nanoribbons with different lengths

S8. Young's modulus of SLMoS_2 nanoribbons by density functional theory (DFT) calculations

S9. Mulliken charges on atoms of Arm-SLMoS_2 nanoribbon

S1. X-ray diffraction of natural MoS_2 block

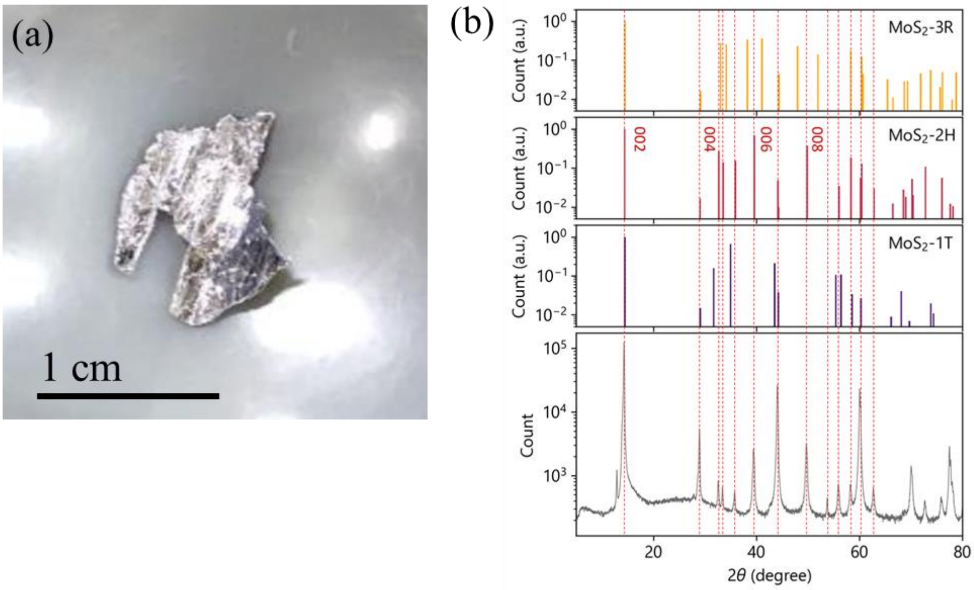

Figure S1. (a) Photographic images and (b) XRD patterns of a block of natural MoS_2.

The natural MoS_2 block is shown in the Figure S1(a), which used for the fabrication of multilayer MoS_2 flakes on TEM grid by exfoliation method. For X-ray diffraction (XRD) measurement, it was crushed to obtain XRD patterns. XRD was carried out using Cu Ka radiation with θ/2θ XRD patterns collected at a scan step of 0.02 deg. and a scan speed of 10 deg./min. The detector used was a D/teX (semiconductor detector).

As shown in Figure S1(b), we find that the peak positions in the experimental XRD match with ones for MoS_2-2H structure, indicating that the natural MoS_2 have MoS_2-2H structure. The different intensity ratio between experimental results and the reference (database) is due to the different sample condition. For the reference data of 1T, 2H and 3R structures, the raw data are obtained by integrating the intensities obtained at all diffraction spots in the reciprocal space using the crystal structure and structure factor determined by measuring these single crystals, not by powder XRD measurements. In other words, they represent the intensity for the case of complete lack of orientation.

S2. TEM image of prepared MoS_2 flake

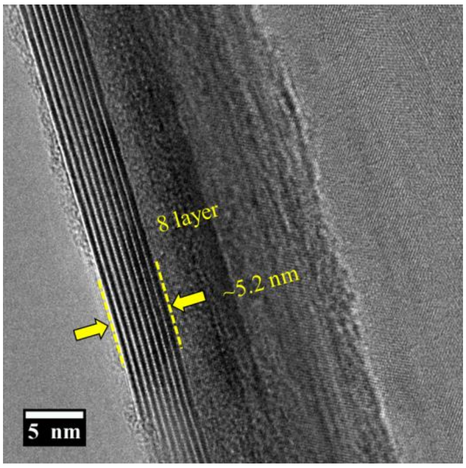

Figure S2. TEM images of folded layers for prepared multilayer MoS_2 flake.

Edge folding of a prepared multilayer MoS_2 flake can be effectively utilized to determine the number of layers.[1] As shown in Figure S2, eight parallel dark lines were observed at the folded edge, indicating that this prepared sample is an eight-layer MoS_2 flake. In addition, the measured thickness of 5.2 nm verified that the layer spacing is 0.65 nm.

S3. Fabrication of the single-layer MoS_2 nanoribbon in TEM

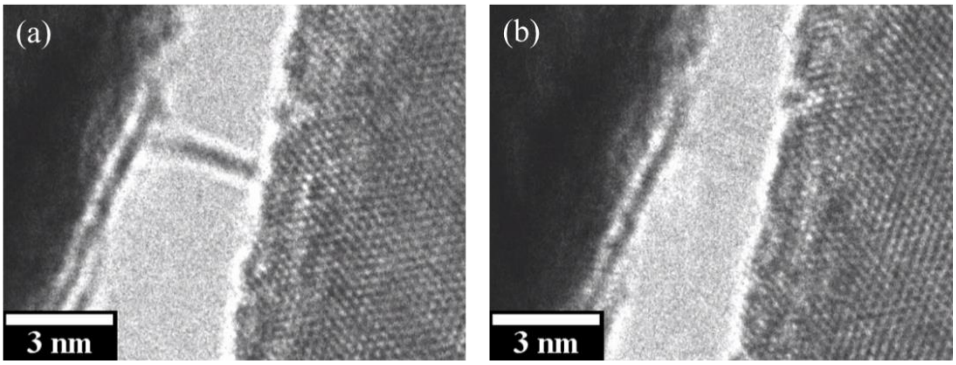

Figure S3. (a) Side-view and (b) plane view of a $SLMoS_2$ bridged between the W-tip and MoS_2 nanoflakes.

The number of layers of the MoS_2 nanoribbon could be checked when it was bridged between the W-tip and MoS_2 nanoflakes, the nanoribbon could be rotated by slightly tuning the position of W-tip. During this process, the number of layers could also be identified through the side-view TEM image of the edge. Figure S3 and Movie S2 shows such process for another $SLMoS_2$.

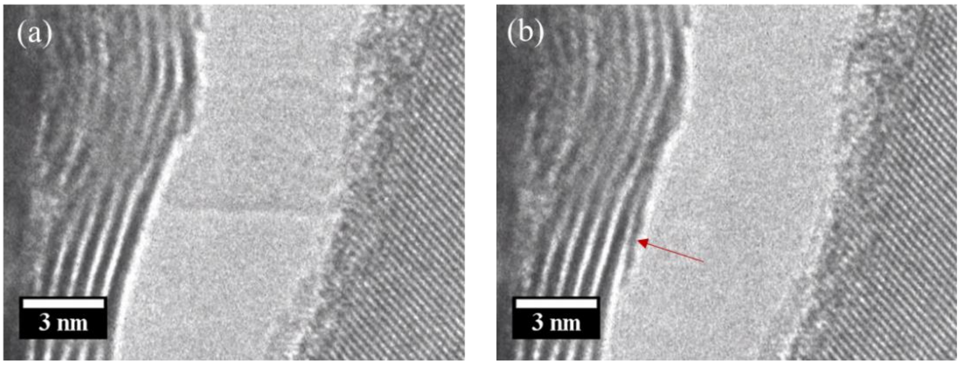

Figure S4. TEM image of $SLMoS_2$ when it attached to one side. Red arrow indicates the edge of the $SLMoS_2$.

Finally, When the mechanical characterization of $SLMoS_2$ nanoribbon was

completed, the W-tip was retracted. Then the $SLMoS_2$ nanoribbon will be detached from either the W-tip or the MoS_2 nanoflakes side and attached on the other side. This process is shown in Figure S4 and Movie S3, which shows the topmost surface layer ($SLMoS_2$ nanoribbon) was retracted after the peel off process, and the layer number also could be identified during this process.

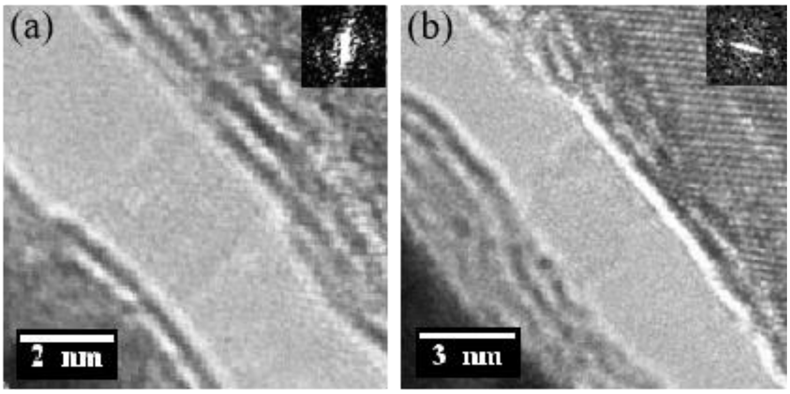

Figure S5. Fabricated $SLMoS_2$ nanoribbons with (a) zigzag and (b) mixed edges. Insets: corresponding FFT patterns.

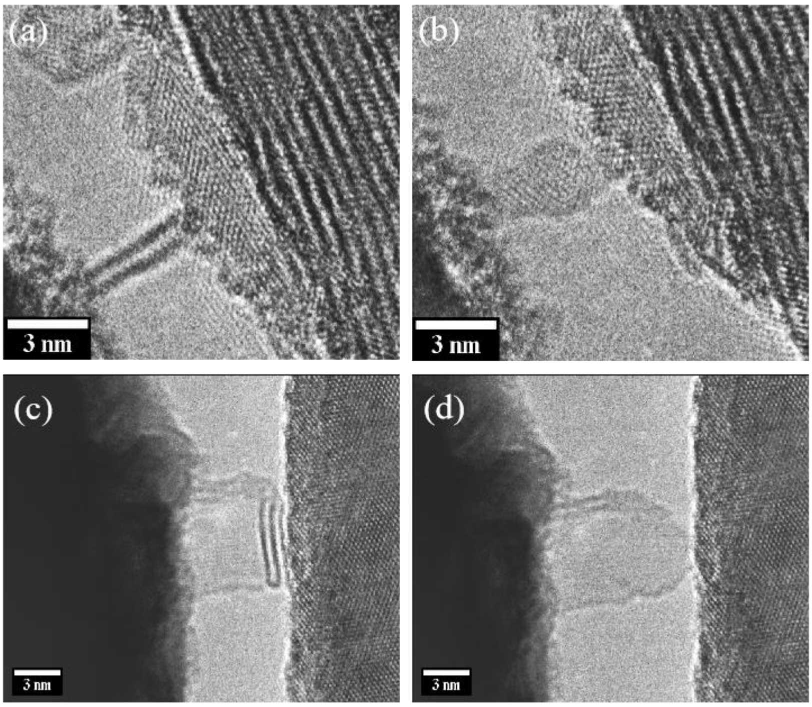

Figure S6. Side-view and plane-view TEM image of (a,b) double layer and (c,d) triple layer MoS_2 nanoribbon.

S4. Developed TEM holder and measurement system

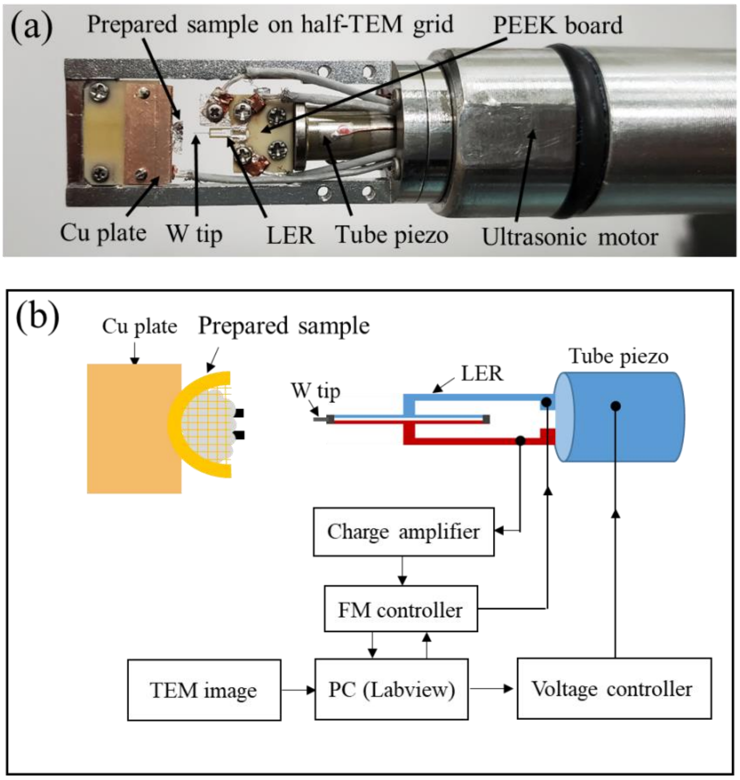

Figure S7. (a) Photographic images of the sample stage at the head of home developed TEM holder. (b) A schematic illustration of the measurement system in the present study.

The photograph of sample stage at the head of home-developed *in-situ* TEM holder are shown in Figure S7(a). On one side, a half-TEM grid with the prepared multilayer MoS_2 flake was fixed to a copper plate using silver (Ag) paste. On the other side, a 10 μm diameter tungsten (W) wire was glued to the head of LER force sensor (3EXW-1073, STATEK), then they were fixed onto a polyether ether ketone (PEEK) board with insulating epoxy resin. For making the electrical excitation of LER, such as inducing LER oscillation and measuring the signal, the two electrodes of LER were electrically connected to two coaxial cables with the Ag paste. The tube piezo and compact ultrasonic linear motor (TULA50, Technohands) was used to control the movement of W wire through fine and coarse motion, respectively.

The measurement system utilized in this work is illustrated in Figure S7(b). By using a phase locked loop (PLL) in a FM controller (OC4, SPECS Zurich GmbH), a sinusoidal excitation voltage was applied to one electrode (colored as blue one) of LER and make the LER oscillated at its resonance frequency (f_0). Then the charge was induced on the other electrode (colored as red one) by the LER oscillation due to piezoelectric effect, and converted to a voltage output signal by a charge amplifier (HQA-15M-10T, FEMTO Messtechnik GmbH, conversion ratio of 10 V pC^{-1}). The frequqncy shift (Δf) was determined by the phase difference between the output and input signals of the LER. The stiffness of the measured MoS_2 nanoribbon (k) was determined by the formula of $k \approx 2 \times k_0(\Delta f/f_0)$, where k_0 represents the stiffness of LER.

S5. Orientation analysis of MoS_2 nanoribbon by FFT pattern

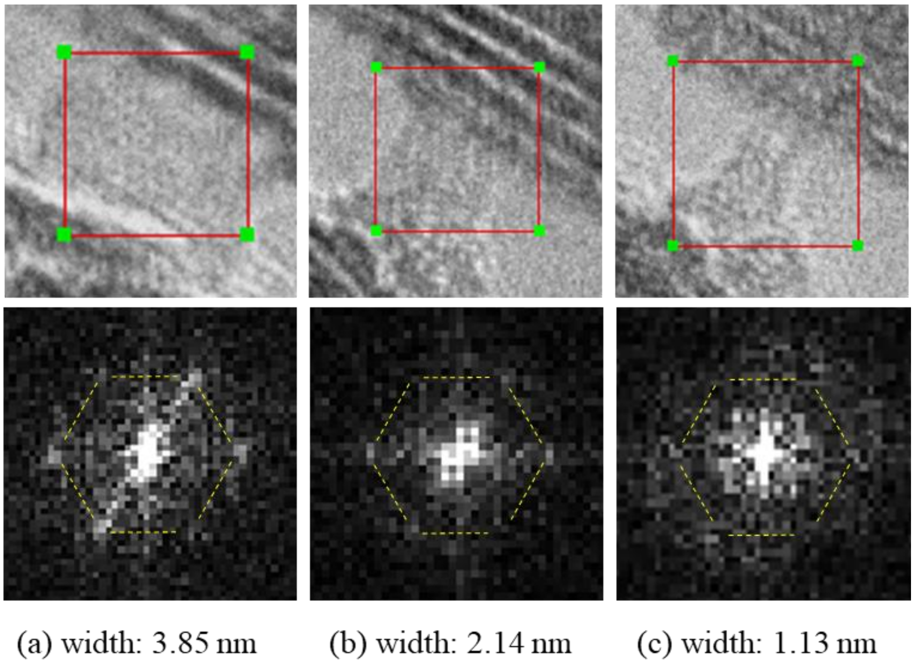

Figure S8. TEM images of armchair edge SLMoS_2 nanoribbons which are the same with Fig. 4(b), (c) and (d) in the main text, respectively, and corresponding FFT patterns.

Six equivalent {100} reciprocal lattice spots can be seen in these FFT patterns. In Figure S8(b), the intensities of the {100} reciprocal lattice spots are weaker than that in Figure S8(a). In Figure S8(c), it is further weaker and somewhat buried in the noise. As the width decreases, the spot intensity becomes weaker and the signal-to-noise ratio becomes worse, making it difficult to obtain the FFT pattern clearly. All three ribbons were fabricated from the same ‘mother’ folded flake. The peeling direction was almost parallel to the armchair edge. The FFT patterns of these TEM images (Figure S8) confirmed that the edge structures were an armchair structure.

S6. Estimation of the stiffness of MoS_2 flake and W tip

For calculating the contributions of the MoS_2 flake and W-tip which support the Arm-SLMoS_2 nanoribbon, we measured their dimensions and calculated the stiffnesses. As shown in Table S1, the size of MoS_2 flake with a thickness of 8 layers was measured from Figure 1(b) in the main paper. The length and width of it was 3.5 and 2.8 μm, respectively. The measured thickness was 5.2 nm as mentioned in the main paper. The Young's modulus used here is 330±70 Gpa, which is the average value of suspended MoS_2 nanosheets with 5 to 25 layers.[2] Accordingly, we calculated the stiffness of the MoS_2 flake (k_{flake}) to be approximately 1373 N/m.

As concerned with the W-tip, since it was impossilbe to accurately estimate its shape, we assumed that the tip was in the form of a cone (hereafter, the cone is referred to as the "connection" part) as shown in Figure S9. And we calculated the stiffness of the W-tip, including the "rod" and "connection" parts as shown in Table S2. The stiffness of the W wire (rod part) (k_W), which has the length and diameter of 126.1 and 10.0 μm, was calculated to be about 2.1×10^5 N/m in Table S1. On the other hand, the stiffness of the connection part depended on the hight of the cone, which was estimated to be 16.2, 5.4, 3.2 and 1.6×10^5 N/m for lengths of 0.1, 0.2, 0.5 and 1 μm, respectively. Therefore, the total stiffness of W tip was 1.9, 1.5, 1.3 and 0.9 $\times 10^5$ N/m for lengths of 0.1, 0.2, 0.5 and 1 μm, respectively. Considering that the aspect ratio of length to wire diameter should be small, we determined that the stiffness of the W tip including the wire was on the order of 10^5 N/m.

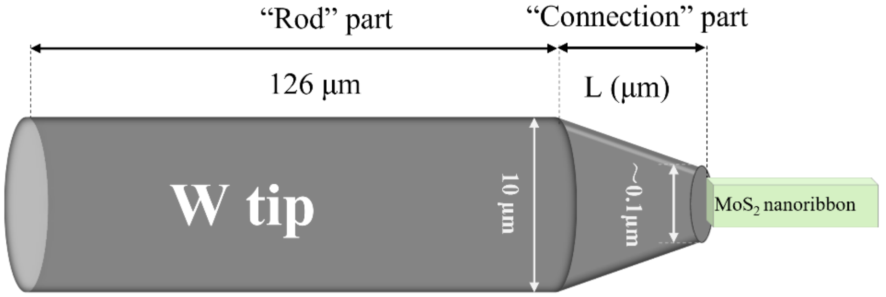

Figure S9. Schematic illustration of the W-tip.

Parameters \ Supporting bases	MoS_2 flake (~8 layers)	Tungsten wire (rod part)
Young's modulus (Gpa)	330±70	340
Length (µm)	3.5	126.1
Width (µm)	2.8	Φ = 10.0 µm
Thickness (nm)	5.2	Φ = 10.0 µm
Stiffness (N/m)	1373	2.1×10^5

Table S1. Parameters of base parts include 8 layers MoS_2 flake and Tungsten wire. The Young's modulus of MoS_2 flake and Tungsten wire are confirmed from reference [2] and [3], respectively.

Length (L) (µm)	Stiffness of Rod (10^5 N/m)	Stiffness of connection (10^5 N/m)	Total Stiffness (10^5 N/m)
0.1	2.1	16.2	1.9
0.3	2.1	5.4	1.5
0.5	2.1	3.2	1.3
1	2.1	1.6	0.9

Table S2. Calculated stiffness of W-tip assuming the cone-shaped tip.

S7. Young's modulus of Arm-SLMoS$_2$ nanoribbons with different lengths

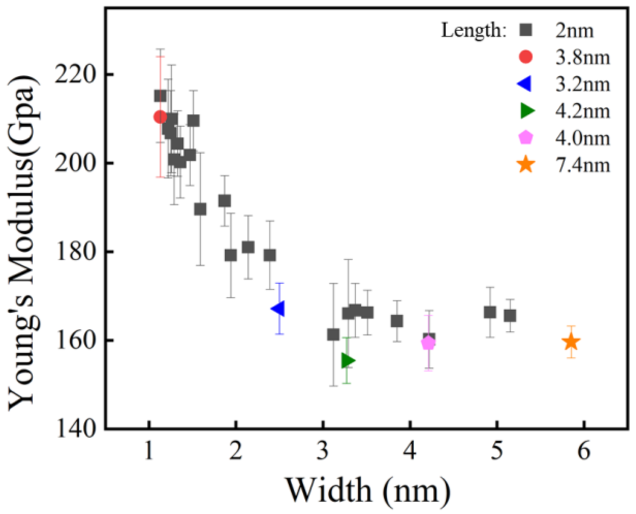

Figure S10. Colorful signals represent the Arm-SLMoS$_2$ nanoribbons with different long lengths.

Figure S10 shows the measured Young's modulus for the Arm-SLMoS$_2$ nanoribbons with 3.2 to 7.4 nm length. Since it was difficult to obtain longer SLMoS$_2$ nanoribbons with perfect armchair edge structures using the peel-off method through cracking[4–6], the longer Arm-SLMoS$_2$ nanoribbons were fabricated occasionally. The longer nanoribbons showed similar Young's moduli with the ~2 nm-long nanoribbons, indicating that the length does not influence the Young's modulus of the nanoribbon.

S8. Young's modulus of $SLMoS_2$ nanoribbons by density functional theory (DFT) calculations

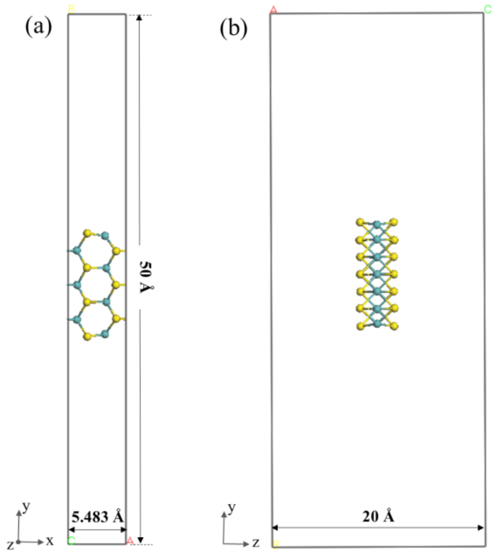

Figure S11. The armchair $SLMoS_2$ nanoribbon with 3 rings in a unit cell from (a) front and (b) side views. The size of simulation cell is 5.483, 50 and 20 Å along the x, y, and z directions, respectively.

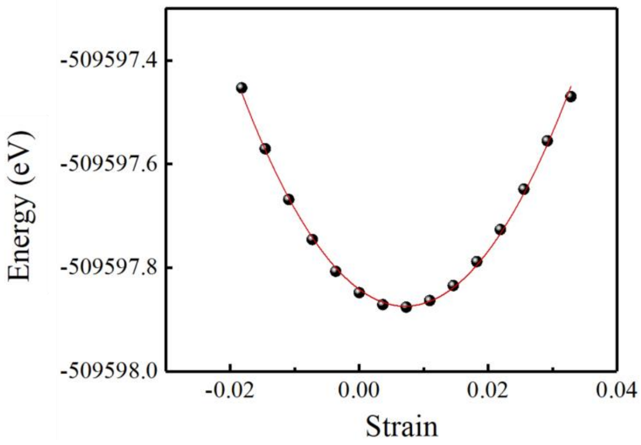

Figure S12. The calculated strain-dependent energy of an armchair $SLMOS_2$ nanoribbon with a width of 3.22 nm.

All of the calculations in this work were carried out using the DMOL3 code.[7,8] The chosen basis set was a double-numerical basis set with polarization functions (DNP),[9] which is confined within a real-space cutoff of 0.49 nm. The DNP is

comparable to Gaussian 6-31G(d,p) and more accurate than a Gaussian basis set of the same size.[9] The exchange-correlation interaction was described within the generalized gradient approximation with the Perdew-Burke-Ernzerhof (GGA-PBE) exchange-correlation functional.[10]

To investigate the stiffness (k) of SLMoS_2 nanoribbon with armchair edges, we assumed the length of ribbon to be infinite in our DFT calculations. For calculating the strain (δ) dependence of energy (E), one armchair SLMoS_2 nanoribbon was placed inside a simulation cell with the following dimensions, as shown in Figure S11: The sizes in y and z direction were fixed to be 50 and 20 Å, respectively. These large vacuum slabs could effectively avoid spurious interactions between repeated images of ribbons due to the periodic boundary condition. The initial size in the x direction was 5.483 Å, which was obtained from the optimized structure. Then we varied the x length from 5.343 to 5.623 Å at a step of 0.02 Å to obtain the strain-dependent energy. In the Brillouin zone of the simulation cell, the 3×1×1 k-point (Monkhorst-Pack k-point) was large enough to obtain converged results for all calculations. Finally, the strain-dependent energy for armchair SLMoS_2 nanoribbon with different width was obtained, shown as in Figure S12.

Young's modulus (Y) of armchair SLMoS_2 nanoribbon is calculated from the second-order derivative of energy with respect to the stain at the minimum. It is defined as:

$$\mathrm{Y} = \frac{\partial^2 \mathrm{E}/\partial \delta^2}{L_0 \cdot w_0 \cdot d_0} \ ,$$

where E is the total energy of supercell, δ is the uniaxial strain applied along the ribbon width direction, L_0 is the lattice constant of the optimized ribbon, W_0 is a finite width of the optimized ribbon, and d_0 is the finite thickness (d_0=0.65 nm) of SLMoS_2 nanosheet. The calculated Young's modulus are shown in Figure 5 in the main paper,

when the width of ribbon is in the range of 0.97 to 4.50 nm.

S9. Mulliken charges on atoms of Arm-SLMoS$_2$ nanoribbon

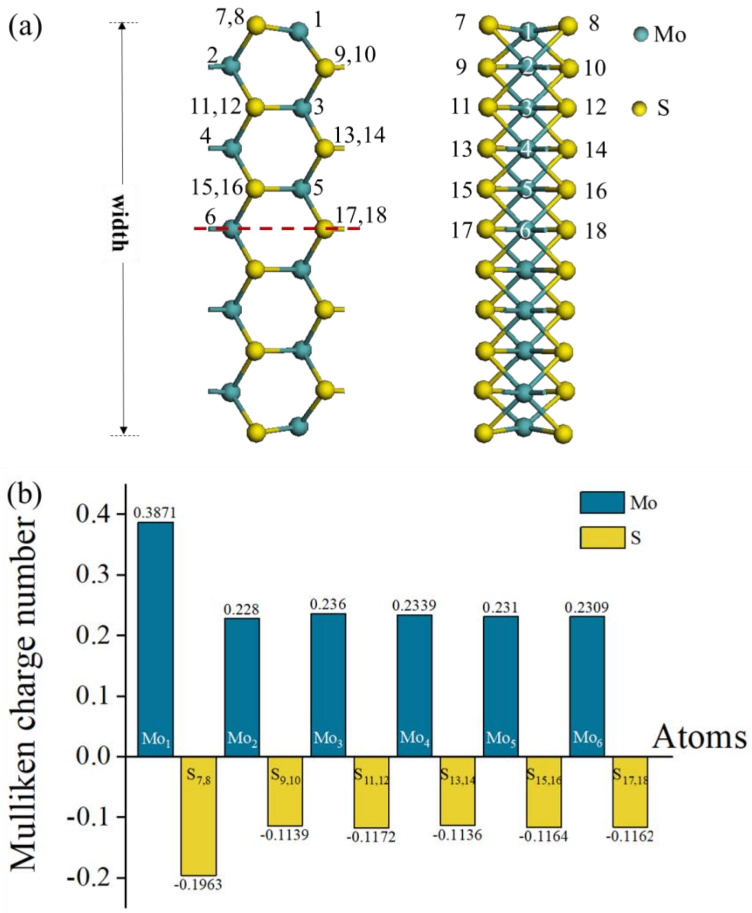

Figure S13. (a) The number of calculated Mo and S atoms of an Arm-SLMOS$_2$ nanoribbon with finite width (5-rings) within a unit cell from the front and side views. (b) Mulliken charge distribution on each atom of Arm-SLMoS$_2$ nanoribbon with 5-rings inside a unit cell.

When calculating the Mulliken charge of each atoms, the calculated Mo and S atoms in a unit cell were numbered, as shown in the front view of Figure S13(a). Since the SLMoS$_2$ nanoribbon has a periodic and symmetric structure, only half of the atoms in the unit cell need to be calculated. The Mo atoms are numbered as Mo_1 to Mo_6 and the S atoms are numbered as S_7 to $S_{18.}$

Individual Mulliken charges at the edge and interior atoms of the Arm-SLMoS$_2$

nanoribbon were calculated for the DFT optimized geometry as shown in Figure S13(a). After optimization, the bond length of Mo-S at armchair edge decreased from 2.424 to 2.312 Å, which is 5% smaller than the original bond. The reducation of the bond length is mainly due to the postion change of Mo_1 atom at armchair edge. However, the bond length of other interior bonds remained mostly unchanged. The Mulliken charge value at each atoms are shown in Figure S13(b). It is obvious that the Mulliken charge values of the edge atoms (Mo_1, S_7) significantly differ from those on interior atoms ($Mo_{2\sim6}$, $S_{9\sim18}$). Thus, we mainly focus on the comparison of Mulliken charge between armchair edge atoms and interior atoms in the main text.

Reference

[1] J. C. Meyer, A. K. Geim, M. I. Katsnelson, K. S. Novoselov, T. J. Booth, S. Roth, *Nature* **2007**, *446*, 60.

[2] A. Castellanos-Gomez, M. Poot, G. A. Steele, H. S. J. van der Zant, N. Agraït, G. Rubio-Bollinger, *Adv. Mater.* **2012**, *24*, 772.

[3] *M.F. Ashby, D.R.H. Jones, Matériaux, Tome I, Dunod, Paris, 1991.*, **n.d.**

[4] G. Wang, Y. Wang, S. Li, Q. Yang, D. Li, S. T. Pantelides, J. Lin, *Adv. Sci.* **2022**, 2200700.

[5] S. Wang, Z. Qin, G. S. Jung, F. J. Martin-Martinez, K. Zhang, M. J. Buehler, J. H. Warner, *ACS Nano* **2016**, *10*, 9831.

[6] K. Kim, V. I. Artyukhov, W. Regan, Y. Liu, M. F. Crommie, B. I. Yakobson, A. Zettl, *Nano Lett.* **2012**, *12*, 293.

[7] B.Delley, J. *Chem. Phys.* 1990, 92 (1), 508–517.

[8] B.Delley, J. *Chem. Phys.* 2000, 113 (18), 7756–7764.

[9] B.Delley,Phys. Rev. B 2002, 66 (15), 155125.

[10] J. P. Perdew, J. A. Chevary, S. H. Vosko, K. A. Jackson, M. R. Pederson, D. J. Singh, C. Fiolhais, *Phys. Rev. B* **1993**, *48*, 4978.
